# HOW ORGANIZATIONS CAN ADDRESS AGEISM AS PART OF THEIR DIVERSITY, EQUITY, AND INCLUSION INITIATIVES AND PROGRAMS

**DOI:** 10.1093/geroni/igad104.2323

**Published:** 2023-12-21

**Authors:** Sasha Perez, Noelle Marie Javier, Katherine Brown, Martine Sanon, Stephanie Chow, Allen Andrade, Nathan Goldstein

**Affiliations:** Icahn School of Medicine at Mount Sinai, New York City, New York, United States; Icahn School of Medicine at Mount Sinai, New York City, New York, United States; Icahn School of Medicine at Mount Sinai, New York City, New York, United States; Icahn School of Medicine at Mount Sinai, New York City, New York, United States; Icahn School of Medicine at Mount Sinai, New York City, New York, United States; Icahn School of Medicine at Mount Sinai, New York City, New York, United States; Icahn School of Medicine at Mount Sinai, New York City, New York, United States

## Abstract

The Brookdale Department of Geriatrics and Palliative Medicine at Mount Sinai Health System created a diverse inter-disciplinary committee to advance and promote DEI activities centered in advocating for a socially just, diverse, equitable, inclusive, and anti-racist workplace. Research shows that anti-ageism is a largely neglected component of diversity, equity, and inclusion programs. Thus, many institutions are not learning how age biases and discrimination show up in the workplace and the intersection of age across other dimensions of diversity. Ageism is a vital workplace issue as over a third (37.3%) of the U.S. workforce are aged 50 and older. We aimed to address this by incorporating the topic of anti-ageism via our various DEI initiatives available to all faculty and staff: “Humanities, Arts, and Books Initiative” on Combatting Ageism - structured workday sessions designed to provide a safe virtual space for reflection, discussion, and learning. We also held “Learning Pathway series” on Reframing Aging as well as Caring for Older Adults with HIV – structured sessions focused on knowledge building, and expert-led discussion. Attendance ranged from 40 to 60 participants at each session. Quantitative and qualitative assessment revealed a steadfast interest to address ageism and bias; appreciation of the semi-structured format for dialogue and reflection on relevant topics and finally ongoing education to create a more informed and collegial department. This type of programming can potentially serve as an additional resource for other institutions that are planning in incorporating anti-ageism activities in their DEI programs and everyday practices.

